# Uncovering the diversity of pathogenic invaders: insights into protozoa, fungi, and worm infections

**DOI:** 10.3389/fmicb.2024.1374438

**Published:** 2024-03-25

**Authors:** Richa Shukla, Jyoti Soni, Ashish Kumar, Rajesh Pandey

**Affiliations:** ^1^Division of Immunology and Infectious Disease Biology, INGEN-HOPE (INtegrative GENomics of HOst-PathogEn) Laboratory, CSIR-Institute of Genomics and Integrative Biology (CSIR-IGIB), Delhi, India; ^2^Academy of Scientific and Innovative Research (AcSIR), Ghaziabad, India

**Keywords:** pathogens, protozoa, fungi, worms, host-pathogen interaction, immune evasion

## Abstract

Post COVID-19, there has been renewed interest in understanding the pathogens challenging the human health and evaluate our preparedness towards dealing with health challenges in future. In this endeavour, it is not only the bacteria and the viruses, but a greater community of pathogens. Such pathogenic microorganisms, include protozoa, fungi and worms, which establish a distinct variety of disease-causing agents with the capability to impact the host’s well-being as well as the equity of ecosystem. This review summarises the peculiar characteristics and pathogenic mechanisms utilized by these disease-causing organisms. It features their role in causing infection in the concerned host and emphasizes the need for further research. Understanding the layers of pathogenesis encompassing the concerned infectious microbes will help expand targeted inferences with relation to the cause of the infection. This would strengthen and augment benefit to the host’s health along with the maintenance of ecosystem network, exhibiting host-pathogen interaction cycle. This would be key to discover the layers underlying differential disease severities in response to similar/same pathogen infection.

## 1 Introduction

The disciplines of agriculture, environmental science, and medicine all greatly benefit from the study of fungi, worms, and detrimental as well as beneficial protozoa. These groups contain pathogenic species that can infect people, animals, and plants; thus, it is essential to study them for strengthening agricultural production and public health. On the other hand, beneficial fungi, worms, and protozoa have a vast range of uses, including the biocontrol of pests, the bioremediation of contaminated environments, and the production of industrial enzymes and pharmaceuticals. The motivations behind the conception of studying both pathogenic and useful organisms lie in the dual pursuit of mitigating health threats and harnessing the beneficial aspects of sustainable development. By understanding the complexities of these microorganisms, researchers will gain information to improve agricultural methods as well as develop focused approaches for disease prevention and treatment, and they will be able to unleash the full potential of these organisms to provide beneficial gains to human health and the environment. This thorough understanding is necessary for advancing fields that directly affect food security, environmental protection, and human health.

## 2 Protozoa

A class of organisms known as protozoa inhabit this hidden domain of extraordinary diversity and complexity where the distinction between microorganisms, plants, and animals is hazy. The Greek words “Protos,” which means first, and “zoa,” which means animals, are the source of the word “protozoa. “Protozoa” are largely free-living creatures that first appeared on Earth about 1.5 × 10^9^ years ago. Protozoa have a highly organized anatomy and carry out sophisticated metabolic processes. It has been noted that these organisms possess a variety of specialized organelles that enable them to move, eat, and reproduce. Some are seen to have whip-like flagella, and others exhibit a kind of locomotion by extracting and retracting pseudopodia (Yaeger, 1996; Zambrano-Villa et al., 2002). Organisms belonging to superior levels of classification hierarchy are observed to be infected with either a single or multiple species of protozoa. Disease conditions may vary subject to the type of the infectious species and can be asymptomatic or in some cases can also be life-threatening. Despite their diminutive size, these microorganisms provide a profound influence on the natural world both as agents of disease and as symbiotic partners (Zheng et al., 2020). Hence, from understanding the mechanism of disease transfer to harnessing the potential of beneficial protozoa the significance of these microscopic organisms extends far from their size.

### 2.1 Protozoa as disease agents

Among a plethora of protozoa, a subset of these microscopic organisms has acquired the ability to infect and further cause disease in humans and animals. These pathogenic strains have evolved complex mechanisms for invasion and survival in the host. Protozoa frequently go through several sequential stages, that provide them the flexibility to take advantage of various tissues or hosts as they make their way through the challenging transition from existence to transmission, and the sickness often results in tissue damage and subsequent illness (Zheng et al., 2020; Akoolo et al., 2022). An assortment of symptoms, from fever and anaemia to cognitive dysfunction and organ failure, can result from the chronic infectious disorders caused by infectious protozoan species that impact the blood, gastrointestinal system, liver, and brain (Seed, 1996). Some of the examples of disease-causing protozoa include *Entamoaeba histolytica, Giardia lamblia* (intestinal parasitic protozoa), *Trichomonas vaginalis* (a urogenital disease in female individuals), *Naegleria fowleri* (meningoencephalitis), *Trypanosoma cruzi* (American Trypanosomiasis or Chagas disease) and *Plasmodium* spp. (Malaria) (Fürnkranz and Walochnik, 2021; Reyes-López et al., 2022) ()thus representing an important set of pathogenic protozoans posing some serious infectious threats among a variety of hosts, including humans.

### 2.2 Mechanism of protozoan pathogenesis by immune evasion strategies and alternative strategies

Protozoa reduce humoral responses, in which antibody–antigen complexes form at the site of excess antibody presence and activate factor XII, also known as Hageman Blood Coagulation Factor, which in turn activates the kinin, fibrinolytic, and complement systems. Additionally, this type of hypersensitivity is linked to several complications, such as edoema, blood hyperviscosity, and hypotension. Similar kinds of humoral immune responses are also observed in other infections caused by protozoa that are listed in Table 1 (Gurung and Kanneganti, 2016; Sardinha-Silva et al., 2022).

**Table 1:** Describing some pathological mechanisms associated with pathogenic protozoa.

Parasitic protozoa	Pathological Mechanism
Malaria	Mechanical tissue damage
African Trypanosomiasis, Malaria	Immunosuppression
African Trypanosomiasis, Malaria	Immediate type hypersensitivity
American Trypanosomiasis	Autoimmunity
Leishmaniasis, Toxoplasmosis, Amebiasis	Delayed type hypersensitivity

In addition to this, some of the most pivotal immune evasion mechanisms utilized by various pathogenic protozoa to infect the host system are majorly observed to be categorized into 5 types:

#### 2.2.1 Intracellular localization

Numerous protozoan parasites proliferate and expand inside the host cells. *Leishmania* and *Toxoplasma*, for example, are known to develop in macrophages, but *Plasmodium* is known to grow in hepatocytes first, then in red blood cells. A portion of the pathogen’s life cycle may be spent inside cells, where it is shielded from both intracellular digestion and the lethal response of lymphocytes and antibodies. For instance, it has been found that the extracellular sporozoite and merozoite stages of the life cycle of the protozoan parasite *Plasmodium* are susceptible to the antibody response. Other than this, it has been noted that *Trypanosoma cruzi* can break free from the phagocytic vacuole and enter the cytoplasm of the macrophage (Sibley, 2011; Graewe et al., 2012).

#### 2.2.2 Antigenic masking

The pathogen encloses itself in the host’s contents during antigenic masking, allowing it to escape and be identified as foreign. For instance, the surface of several species of trypanosomes is attached to host immunoglobulins. These immunoglobulins block the parasite from being recognised by the host molecules’ immune systems because they are not linked to the variable region of the molecule, most likely through the Fc fragment (Chulanetra and Chaicumpa, 2021).

#### 2.2.3 Blocking

**Figure 1:**
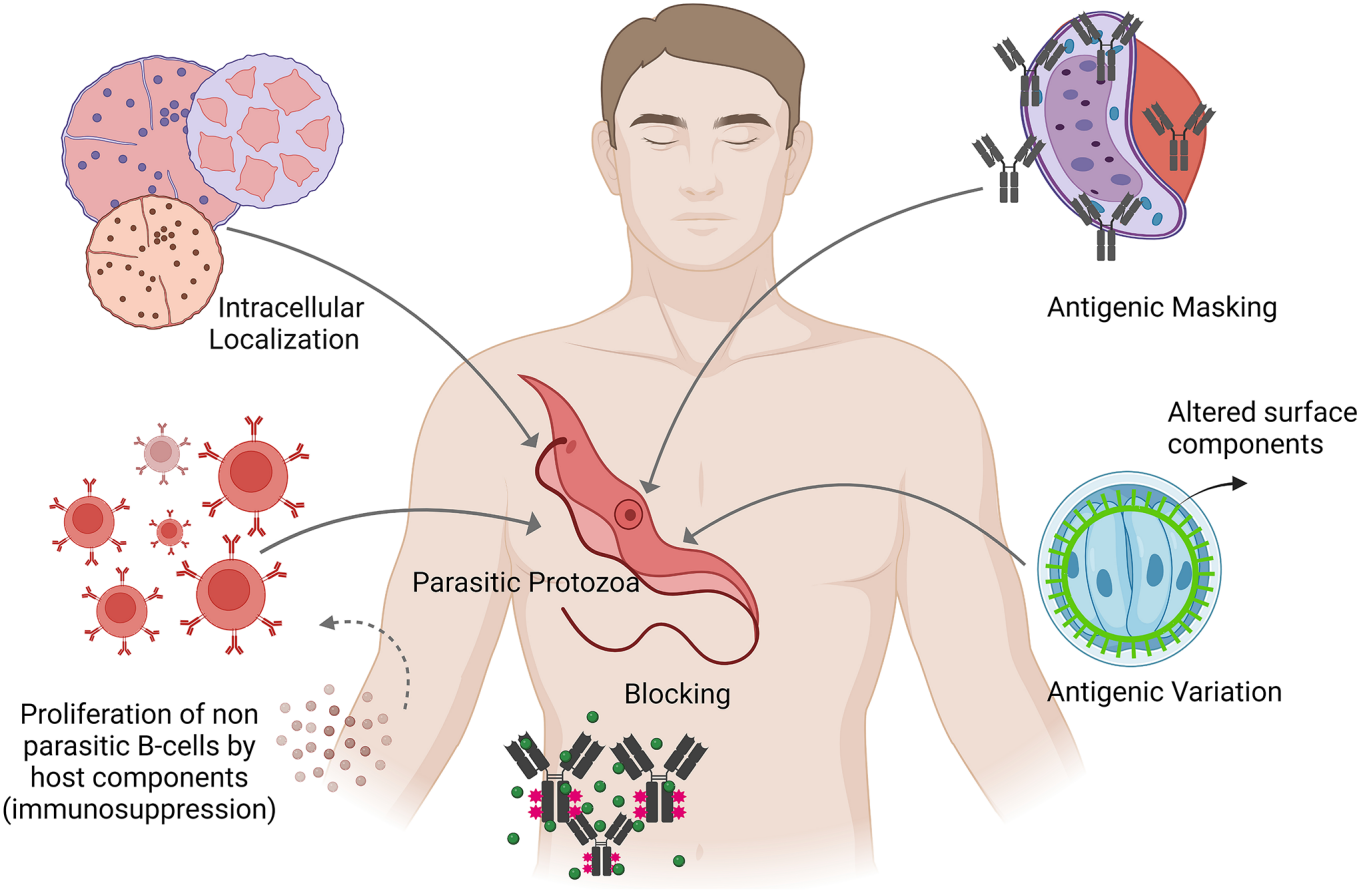
Depicts the various immune evasion strategies employed by the pathogenic protozoa inside the human host.

When antigen–antibody complexes in the serum of infected animals attach to the surface of the pathogen, they impede the pathogen’s ability to elicit a response from lymphocytes and cytotoxic antibodies, so precisely limiting the activity of lymphocytes directed towards the pathogenic organism (Chaplin, 2010). This kind of immunological escape mechanism has been proposed for parasite helminths and cancer cells. Figure 1 depicts the various immune evasion strategies employed by the pathogenic protozoa inside the human host.

#### 2.2.4 Immunosuppression

In immunosuppression, the host’s immune activity is lowered either towards a pathogen specifically or in a generalised manner towards a foreign antigen. With context to protozoan infections, a number of mechanisms have been proposed that include: (i) The presence of the host components that non-specifically activate the proliferation of specific anti-parasitic B cells. (ii) Production of a particular immune suppressor component by the pathogen. (iii) The generation of regulatory cytokines by suppressor T-cells or macrophages, which impair the immune system (Dupont et al., 2012; Saleki et al., 2023).

Due to immunosuppression, a few microorganisms may be able to evade the immune system and develop a persistent infection. Because it allows for the undiscovered presence of a few parasites bearing novel surface antigens, this kind of mechanism may be useful for infections undergoing antigenic change (Finlay and McFadden, 2006). Experimentally induced immunosuppression by a number of external components has been observed to produce elevated parasitism, higher infection rates or both. Other than this, immunosuppression is taken to be pathogenic itself as it has been observed that a lowered response towards heterologous antigens could be advantageous for secondary infections and in turn these infections may often be involved in death as seen in the case of *African trypanosomiasis* (Evering and Weiss, 2006).

#### 2.2.5 Antigenic variation

During an infection, some pathogenic protozoans change their surface antigens, a process known as antigenic variation, which helps them avoid being recognised by the host’s immune system. It has been determined that *Babesia*, *Plasmodium*, and *Giardia* are three significant groups of pathogenic protozoa that can change the antigenic characteristics of their surface coat (Dzikowski and Deitsch, 2006; Chulanetra and Chaicumpa, 2021). For example, *African Trypanosomes* can fully substitute the antigens on their surface glycocalyx every time the host exhibits a new humoral response. It has been documented that approximately one thousand genes encoding surface antigens can be found in African trypanosomes. For these genes to be active, they must be positioned in the telomeric region of the chromosome, even though they are located on various chromosomes. Furthermore, a multitude of separate gene families that encode outer surface proteins in *Giardia* have been identified and suggested to aid *Giardia* in evading the immune response of the host through the mechanism of antigenic diversity (Nash, 1997; Faubert, 2000; Lee et al., 2021). The apicomplexan parasites of the genus *Plasmodium* develop in erythrocytes in repeated cycles during malaria infection. In addition to variations in antigenic diversity within a strain, the species *Plasmodium falciparum* exhibits strains that differ in many polymorphic proteins. The *Plasmodium falciparum*-infected erythrocyte membrane protein 1 (PfEMP1) antigens, which are expressed on the surface of the infected erythrocytes, are the finest known example of genuine antigenic variation. Through altering the expression of PfEMP1, the parasite circumvents the immune reaction aimed at these immuno-dominant antigens. The PfEMP1 proteins also prevent dendritic cells from presenting antigens and enable infected red blood cells to adhere to the endothelium and extracellular matrix, preventing the spleen from eliminating the infected erythrocytes. PfEMP1 variable proteins have a molecular weight between 200 and 350 kDa. A range of endothelial receptors, including CD36, vascular cell adhesion molecule-1 (VCAM-1), E-selectin (ELAM-1), and intercellular cell adhesion molecule type 1 (ICAM-1), as well as the extracellular matrix protein thrombospondin, can be found in their extracellular region. These variable adhesive domains give the parasite-infected erythrocytes a specific binding specificity. The clinical signs of malaria are determined by the adhesive characteristics that cause infected erythrocytes to be sequestered in the liver, kidneys, brain, lungs, or other organs.

Similar to *Plasmodium*, *Babesia* is an intraerythrocytic parasite that is spread by ticks rather than mosquitoes. Clonal antigenic variation of the bovine parasite *Babesia bovis* is the most reported variation, even though multiple multigene families have been described for different species of *Babesia*. The surface of infected red blood cells expresses a heterodimeric protein known as the variable erythrocyte surface antigen (VESA1) of *Babesia bovis*. These polymorphic proteins very quickly, which probably prolongs the parasite’s life through immune evasion and sequesters the infected red blood cells in peripheral organs, leading to chronic infection in cattle. With an estimated molecular weight of 128 kDa, the VESA1 proteins are expressed on the outermost tips of the membrane knobs in infected erythrocytes. The VESA1 proteins’ structural alterations and antigenic modifications determine their cytoadhesive behaviour (Barbour and Restrepo, 2000).

#### 2.2.6 Prevention of phagocytosis

By altering its interaction with host phagocytic receptors as well as controlling downstream signaling cascades, *Plasmodium* species inhibits phagocytosis. For instance, *Plasmodium yoelii* primarily infects erythrocytes that express high levels of CD47 (marker responsible for preventing phagocytosis), thereby helping them avoid being phagocytosed by the splenic red pulp macrophages. Additionally, through modifications to complement regulatory proteins that insulate infected host cells from complement-mediated damage, parasites prevent phagocytosis. They deactivate C3b on the surface of infected erythrocytes, impeding complement-mediated phagocyte removal of the parasites (Cristina Vanrell and Silvia Romano, 2023).

#### 2.2.7 Resistance to oxidative response

In the case of *Trypanosoma cruzi*, reduction of ONOO synthesis in NO-exposed parasites, regulation of NO-exposed parasites, and protection from the direct lethal effects of O2/H2O2 on parasite mitochondria within the macrophage phagosome are few of the strategies that the parasite employs to resist the deleterious effects of oxidative responses from the host. *T. cruzi* possesses a plethora of detoxifying antioxidant defenses and redox metabolism for protection against host-derived oxidants. One of the most significant thiols utilized by the trypanosomatid-antioxidant system is trypanothiol (T[SH]2). Additionally, *T. cruzi’s* Fe-dependent superoxide dismutases (Fe-SODs) efficiently eliminate O2 and may aid in intracellular survival (Estrada et al., 2018). *T. cruzi* has also been reported to harbour TcAPxCcP, a type A hybrid peroxidase that uses cytochrome C and ascorbate as reducing substrates for H2O2 elimination. TcAPxCcP is a membrane-bound peroxidase that is present in the mitochondria and endoplasmic reticulum as well as plasma membrane during the parasite’s life cycle (Hugo et al., 2017). On the whole, *T. cruzi’s* antioxidant defense mechanism detoxifies reactive species in the phagosomal compartments, which contributes to its virulence.

#### 2.2.8 Formation of a distinct vacuole to hinder host defense

In this case, the organelle harboring *Toxoplasma gondii* appears to be arrested and unable to merge with lysosomes, hence protecting host-defense system and successful survival of the parasite. Apart from this, *T. gondii* demonstrates ways by which intracellular pathogens employ certain components of the host pathway (for instance Rab-family GTPases) for nutrient transportation to promote the proliferation of the pathogen (Robibaro et al., 2001; Paone and Olivieri, 2022).

#### 2.2.9 Uptake of hemoglobin from the host

Hemoglobin and ferritin (specific intracellular proteins) are the main cellular reserves of iron in mammals. Erythrocytes contain hemoglobin (Hb) which is a vital supply of iron and amino acids for pathogenic protozoa to flourish inside the infected host. For instance, in the case of *Giardia lamblia* (an intestinal protozoan parasite), the parasite is observed to exhibit lysosomes as peripheral vacuoles with hydrolase and acid phosphatase activity that might be pivotal in cell feeding and excystation. The ideal pH range for these proteinases to function against Hb is 3.5 to 7.0; hence, protease secretion directed against Hb influences host interactions. In addition to this, in the event of *Trichomonas vaginalis*, a certain percentage of the reason trichomonads are successful parasites of the vaginal epithelium is linked to their ability to take in vital nutrients like iron. Since, in addition to controlling cytoadherence, cytotoxicity, hemolysis, complement resistance, immunological evasion, and apoptosis in human cells, iron likewise impacts the virulence of *T. vaginalis*. As there is no free iron in the vaginal environment, *T. vaginalis* obtains iron from host proteins that either bind to iron or contain iron, including cytochromes, Lactoferrin, and Hb. When the amount of lactoferrin decreases during menstruation, hemoglobin (Hb) in erythrocytes serves as a significant source of iron. Hemolysis and erythrophagocytosis are the two main methods that *T. vaginalis* uses to obtain Hb iron *in vivo*, which serves to aid in the growth and differentiation of the parasite inside the host (Reyes-López et al., 2023).

### 2.3 Protozoan diseases in humans

#### 2.3.1 African trypanosomiasis (sleeping sickness)

Human African trypanosomiasis (sleeping Sickness) is a vector-borne parasitic disease. The disease is transmitted to humans by bites of *Glossina* (tsetse fly) which have acquired the disease-causing protozoa from infected animals or humans. Pathogenic protozoa take two forms depending on the parasite subspecies called as *Trypanosoma brucei gambiense* and *Trypanosoma brucei rhodesiense*. With a tiny kinetoplast and a well-developed undulating membrane, these two *T. brucei* subspecies are morphologically very similar. While in the blood or cerebral fluid, parasitic protozoa multiply. A feeding fly ingests trypanosomes, which then enter the salivary glands and reproduce rapidly as epimastigotes attach to the gland’s microvilli until they modify into metacyclic trypomastigotes found in the lumen. About 15 to 35 days after infection, the fly becomes contagious for humans. The disease is mainly transmitted through tsetse fly however there are other pivotal possible ways of disease transmission like: (i) Mother to child: Trypanosomes can cross the placental barrier and hence can infect the foetus. (ii) Infection in laboratories via pricks with contaminated needles. (iii) Transmission through sexual contact.

##### 2.3.1.1 Clinical features

With context to the clinical attributes associated with the disease the parasite is initially observed to be multiplying in lymph, blood, and subcutaneous tissue, called the hemo-lymphatic stage (first stage), leading to symptoms like headache, fever, itching, joint pain, and enlarged lymph nodes. Following this, the pathogen crosses the blood–brain barrier and enters the nervous system, causing the meningo-encephalitic stage (second stage), which displays signs including sensory commotion, behavioural changes, sleep-cycle disturbance, and poor coordination. In addition to this, when a patient has *Trypanosoma brucei rhodesiense* infection, they often have an early symptom of regional lymphadenitis; however, this is less common in people with *T. b. gambiense* infection. Following a phase of localized growth, the trypanosomes penetrate the bloodstream through the lymphatic system, potentially causing recurrent fever, headaches, lymphadenopathy, and splenomegaly. Subsequently, somnolence, cachexia, coma, and death occur, along with indications of meningoencephalitis. Enlargement of the posterior cervical chain of lymph nodes, also known as Winterbottom’s sign, is more frequently caused by a *T. b. gambiense* infection (Kennedy and Rodgers, 2019).

##### 2.3.1.2 Pathogenesis and host defense

In order to survive in hosts that are continuously infected, African trypanosomes have developed incredibly complex evasion strategies, including antigenic mutations, excessive complement system activation resulting in chronic hypocomplementemia, downregulation of nitric oxide generation, polyclonal B-lymphocyte activation, and severe immunosuppression (Bezie, 2014). Inflammatory changes leading to demyelinating encephalitis are observed as the disease progresses. Associative infections like pneumonia have been proposed to be caused by the parasite’s membrane’s immunosuppressive response. In addition to this, the liberation of common antigens in each episode of trypanolytic crisis (trypanosome lysis) leads to antibodies as well as cell-mediated hypersensitivity reactions. At the site of inoculation, inflammatory reactions occur, along with regional lymphadenitis. Inflammation in the brain and heart also develops, accompanied by a cell-mediated immune reaction along with higher levels of IgM.

##### 2.3.1.3 Diagnosis and control

The pathogen can be found in the blood and lymph nodes of the human host infected early in the disease’s course, and subsequently in the host’s cerebrospinal fluid. The Gambian form of the infection appears to be persistent and takes years to develop CNS-related abnormalities, whereas the Rhodesian variant develops more acutely and eventually results in death. For the proper disease diagnosis laboratory animal inoculations or cultures are found to be useful. Apart from this, serological tests including indirect hemagglutination and indirect immunofluorescence are also found to be advantageous for disease detection. In terms of control methods, pesticides and traps have been used to regulate tsetse fly populations. Apart from this, medications like melarsoprol and eflornithine (difluoromethyl-ornithine) are used to treat severe stages of illness conditions, while pentamidine and arsenical suramin are used to treat early phases of infection.

#### 2.3.2 Trichomonas vaginalis

*Trichomonas vaginalis* is a pathogenic protozoan responsible for causing trichomoniasis, i.e., the most widespread, non-viral sexually transmitted infection (STI). *T. vaginalis* is a motile protozoan species that inhabits the lower genitourinary tract of females and prostate and urethral regions in males (Petrin et al., 1998; Menezes et al., 2016). The concerned disease is observed to elevate the risk of Human Immunodeficiency virus (HIV) transmission in both males and females. Other than this, the disease is also linked with detrimental outcomes during pregnancy. The pathogen possesses a pear-shaped body organization, a single anterior nucleus and a single posterior flagellum that constitutes the exterior border of an undulating membrane. The life cycle of *Trichomonas* is quite uncomplicated as it remains only as a single trophozoite form and since there is the absence of any resistant cyst-like stage the mode of transfer from host to host is simply direct. The various types of risk factors associated with the infection (Beri et al., 2019). It includes association with an infected partner, multiple sex partners, person associated with any history of Sexually Transmitted Infections (STIs), drug abuse, and avoiding usage of any barrier contraception.

##### 2.3.2.1 Clinical Features

**Figure 2:**
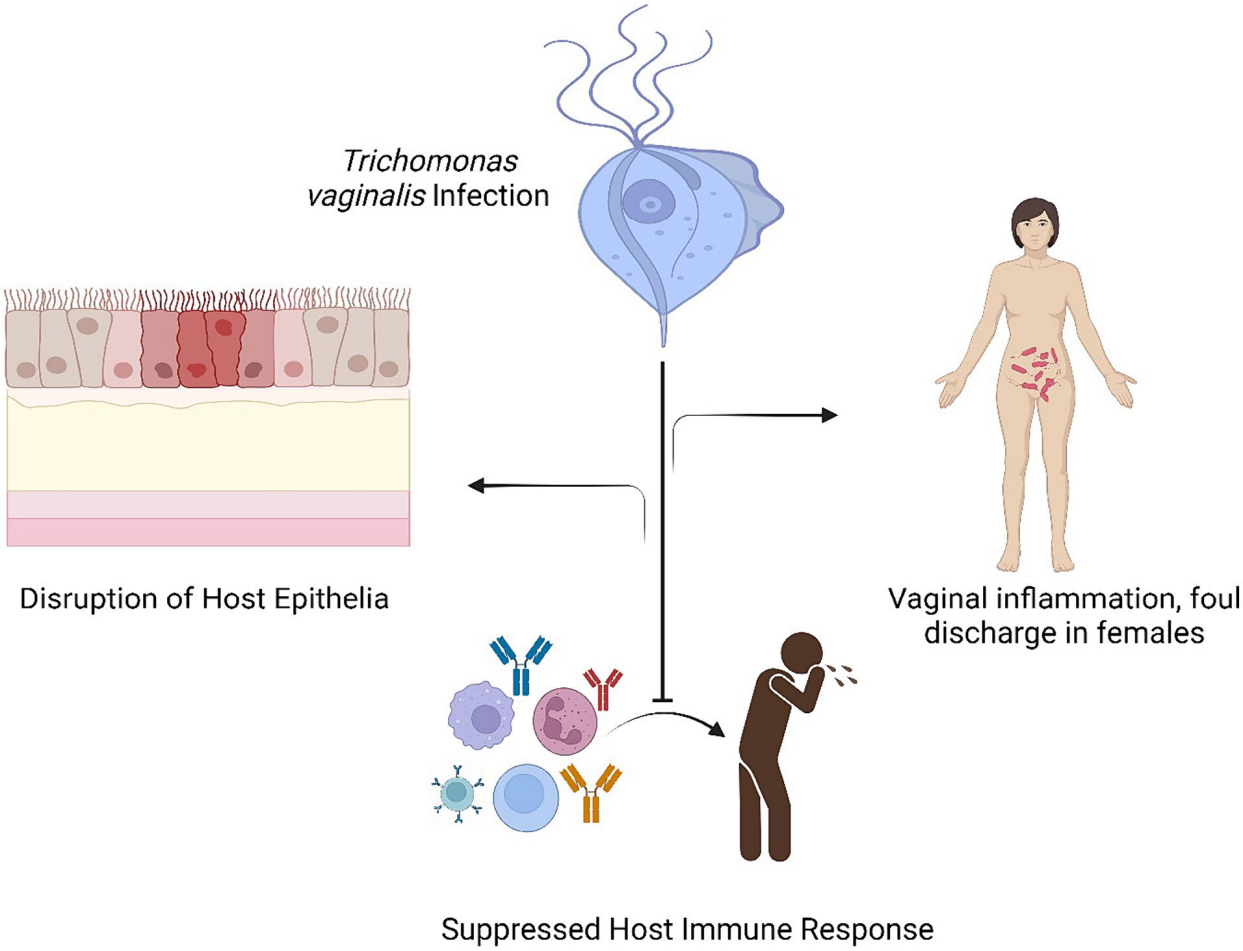
Interprets the various associated outcomes with response to *Trichomonas vaginalis* infection.

*Trichomonas* infection in females is generally chronic and is represented primarily by dysuria, vaginitis and vaginal discharge The swelling of the vagina is generally spread out and is represented by hyperemia of the vaginal wall along with the movement of polymorphonuclear leukocytes into the vaginal lumen Most women (85%) and men (77%), who are infected with *Trichomonas vaginalis*, do not exhibit any symptoms. In less than 6 months, one-third of asymptomatic women develop symptoms. The urethra, the vagina, and the endocervix are common places for infections in women. The pH of the vagina is normally 4.5, but when there is a *Trichomonas vaginalis* infection, it frequently rises to >5. Approximately, 5% of women experience *coplitis macularis*, also known as strawberry cervix; however, this number increases to almost 50% following colposcopy. Adnexal, endometrial, Skene and Bartholin gland infections are among the other side effects. *Trichomonas* infection can result in decreased sperm cell motility, prostatitis, and epididymitis in males (Kissinger, 2015). Figure 2 interprets the various associated outcomes with response to *Trichomonas vaginalis* infection.

##### 2.3.2.2 Pathogenesis and host defense

Regarding the pathogenesis of *Trichomonas vaginalis*, all pathogenic mechanisms (i.e., immune response, contact-dependent, and contact-independent) are likely relevant in the virulence of this illness. Four adhesion proteins – AP65, AP51, AP33, and AP23 – seem to be involved in the parasite’s attachment to epithelial cells. Their specific receptor-ligand interactions are influenced by pH, temperature, and time. The host cell receptors that the parasite adhesion molecules attach to are not well understood; however, there is some indication that laminin may be a potential target for trichomonad adherence. Conversely, it seems that the parasite’s virulence and hemolytic activity are connected. Considering that *T. vaginalis* is incapable of synthesizing lipids, erythrocytes could be a major supply of the fatty acids the parasite requires. Furthermore, the lysis of red blood cells might provide the parasite with iron, a crucial nutrient. Moreover, a wide range of hydrolases, with cysteine proteinases being particularly common, have been identified in *T. vaginalis*. Apart from this, *Trichomonas* symptoms are frequently worse during menstruation; this fact may be explained by the functions that pH and hormones play in the disease. Menstrual blood raises the pH above what is typically found in the vagina, providing a rich environment for *T. vaginalis* proliferation. Furthermore, more iron is made available by the blood, which improves *T. vaginalis’* capacity to adhere to the vaginal epithelium (Preethi et al., 2011). Additionally, the reason for the inflammation reaction in the case of trichomoniasis is not completely clear, however, one of the reasons suggested for the condition is mechanical irritation occurring from contact between the vaginal epithelium and the parasite. Research investigations using animals suggest the protective role of antibodies in overcoming signs and symptoms associated with the infection. However, the antibody response activated during the infection is short-lived and disappears completely in 6 to 16 months (Zhang et al., 2023).

##### 2.3.2.3 Diagnosis and control

Typically, a wet mount preparation of discharge from the patient is tested for diagnosing *T. vaginalis* infection. The presence of pear-shaped trophozoites, typically 7 to 23 μm in length with “bobbling.”

#### 2.3.3 Malaria

Malaria is a parasitic disease transmitted by *Anopheles* mosquito that causes acute life-threatening condition and poses a serious global health threat. The multistage developmental process of *Plasmodium* causes the infected host to have periodic fever bouts. With consideration to the life cycle of the pathogenic protozoan, *Plasmodium* undergoes two stages of development: an asexual stage in the human host and a sexual phase in the *Anopheles* mosquito, which serves as the carrier. Within the liver parenchymal cells, where they proliferate asexually, the sporozoites that are delivered during a blood meal quickly enter the bloodstream (a phase called as schizogony). The asexual reproduction of protozoa is known as schizogony, previously known as merogony. A schizont is made up of many cell nuclei; the daughter nuclei organize into single entities called merozoites when they are encircled by cytoplasm. Between 10,000 and over 30,000 merozoites can be produced by a single sporozoite. The enlarged liver cell bursts and discharges the mobile merozoites into the bloodstream when schizogony gets completed. These merozoites stick to the red blood cells through certain surface receptors (glycophorin A in the case of *P. falciparum*, or Duffy blood group antigen, Fya or Fyb, in the case of *P. vivax*). Following their entry into red blood cells, they develop into trophozoites. By the time the 48-to 72-h erythrocytic phase concludes, the red blood cells’ schizonts get developed. Vacuoles with parietal nuclei, often known as seal-ring forms, might develop during this period. Red blood cells that have degraded may release fresh merozoites that can infect more red blood cells. Within erythrocytes, a portion of the merozoites evolves into sexual phases, creating macro-and microgametocytes. The malaria pigment, an insoluble metabolite of hemoglobin, forms hemozoin in the intra-erythrocytic vacuoles. The *Anopheles* mosquito’s midgut produces a motile, flagellated zygote following the uptake of both male and female gametocytes during a blood meal. This zygote enters the gland that produces saliva. Finally, an oocyst forms, generating sporozoites that can spread to a new human host through the mosquito’s saliva (Arbeitskreis Blut, Untergruppe «Bewertung Blutassoziierter Krankheitserreger», 2009; Cox, 2010; Venugopal et al., 2020).

##### 2.3.3.1 Clinical features

The most common symptom associated with the infection is fever. Other major signs related to malaria include vomiting, nausea, myalgias, diarrhea, chills, abdominal pain and headache. With the advancement of the disease, some cases are observed to develop the classic malaria paroxysm that consists of 3 successive stages:

- Stage 1 (Cold stage): 15–60 min characterized by a feeling of cold and shivering.
- Stage 2 (Hot stage): 2–6 h consisting of fever (around 41°C), nausea, vomiting, flushed dry skin and headache.
- Stage 3 (Sweating stage): 2–4 h. Fever drops promptly and the patient sweats (Nureye and Assefa, 2020).

In addition to this, various other important complexities associated with the infection include cerebral malaria, nephrotic syndrome (NS) and severe malarial anaemia. Other than this some pivotal added complications associated with malaria are:

- Algid Malaria, an adrenal deficiency that occurs due to pathogenic blockage and associated necrosis of the adrenal gland.
- Bilious remittent fever characterized by abdominal pain and constant vomiting that may cause jaundice, dark urine and dehydration.
- Circulatory collapse, acute respiratory distress syndrome, pulmonary edema, intravascular coagulation, coma and death (Vandermosten et al., 2018).

In an instance of simple malaria, several laboratory abnormalities could be observed. The conditions that fall under this category are proteinuria, increased liver and renal function tests, thrombocytopenia, leukocytosis or leukopenia, hypoglycemia, hyponatremia, and laboratory evidence of disseminated intravascular coagulation. Rarely, patients with severe malaria may exhibit hemoglobinemia and hemoglobinuria together with significant intravascular hemolysis. Additional risks include shock, pulmonary, cardiac, hepatic, or renal failure, convulsions, hyperparasitemia (greater than 3–5% of the RBCs get parasitized), prolonged hyperthermia, severe hypoglycemia, lactic acidosis, spontaneous bleeding, high output diarrhea or vomiting. Splenic rupture, aspiration pneumonia, and gram-negative sepsis are further malarial infection consequences (Idro et al., 2005).

##### 2.3.3.2 Pathogenesis and host defense

Clinical symptoms are associated with the erythrocytic stage of the pathogen. Primary symptoms of malaria are associated with erythrocyte rupture, which sets off the host’s immunological response. Reactive oxygen intermediates, cytokines, and other substances generated during immunological activity are thought to be important players in pathogenesis, causing fever, chills, weakness, and other infection-related systemic symptoms. With context to *Plasmodium falciparum*, infected red blood cells stick to the endothelium of capillaries and post-capillary venules causing blockage of microcirculation and local tissue anoxia. In other organs including the kidney, intestine and brain the pathogen is observed to be responsible for causing acute tubular necrosis, ulceration and cerebral malaria, respectively (Clark et al., 2004; Joos et al., 2010). Apart from this, the pathophysiological basis of severe malaria manifestations, including brain malaria, is believed to be parasite sequestration. It impairs blood flow, which results in localized hypoxia. It promotes the growth of parasites and the ability of infected RBCs to adhere to healthy red blood cells. Furthermore, when parasites sequester, the effects of their toxins are more restricted, and the host immune response is also stimulated, perhaps leading to targeted synthesis of inflammatory mediators and tissue damage. RBC and infected RBC thus become less malleable and stiffer. Sequestration occurs when parasites attach themselves to the placenta during gestational malaria. The primary adhesion receptor, *P. falciparum* erythrocyte membrane protein 1 (PfEMP1), binds to the trophoblastic villous endothelium mostly through chondroitin-4-sulfate (CSA) and other sugars such as glycosaminoglycans as well as hyaluronic acid (HA). In particular, during the first pregnancy, when women typically lack adequate antibodies against CSA-binding parasites, malaria during pregnancy can be extremely dangerous for mothers and result in fetal death (Autino et al., 2012). In view of the host response, malaria is proposed to be regulated by acquired as well as hereditary factors. For example, the lack of *P. vivax* infection in African regions can be explained through the absence of Duffy-blood group antigens, which *P. vivax* merozoites bind to. Ovalocytosis, which is common in some malaria-prone areas like New Guinea, has been suggested to potentially lower the incidence of malaria since malarial infections do not grow in ovalocytes. Aside from this, cytotoxic T-cell response, which is used to counteract the pathogen’s liver stages, and naturally developed immunity, which involves cytokine release, both function to guard against all stages of the infection (Rodrigues et al., 2014).

##### 2.3.3.3 Diagnosis and control

Direct pathogen observation in thick and thin blood smears stained with Giemsa is typically required for a specific diagnosis of malaria. Thin blood smears in which the parasite is visible inside the red blood cells are used to suspect the species of the infective pathogen. Advanced diagnostic methods that are quite specific, fast, sensitive and easy to perform include a rapid antigen-capture dipstick test as well as the use of a fluorescent stain in order to identify the parasite (Fitri et al., 2022). Other than this, some of the methods that are majorly used in epidemiologic investigations and immunization trials include assays to identify malarial antigens and antibodies and polymerase chain reaction (PCR) DNA/RNA probe techniques. The various control measures that can be taken up for proper management of malaria include early detection of infection, rapid establishment of suitable therapy, and examining the clinical and parasitological response to therapy (Moody, 2002).

Therapy options related to malaria are quite complex as the pathogen can be found in the liver and blood of the infected host and thus various drugs are needed to eliminate each. Drugs that eradicate infections in the bloodstream are referred to as blood-stage schizonticides, whereas those that eradicate them in the liver are known as tissue schizonticides. Treatment options include combination therapy addressing both the erythrocytic and hepatic forms. The major antimalarials include hydroxychloroquine, chloroquine, primaquine and artemisinin-based combination therapy (ACT). Hydroxychloroquine and chloroquine disturb the erythrocytic stage by disrupting the hemoglobin metabolism of the pathogen and elevating the intracellular pH. Artemisinins remain therapeutically functional against all life cycle stages of malaria (Deroost et al., 2016; Bhattacharjee et al., 2023).

### 2.4 Beneficial role of protozoa

#### 2.4.1 Role of human protozoa in molecular therapy (symbiosis therapy)

Symbiosis with its literal meaning as “living together” is defined as an organism that survives in a group and provides each other collective benefits. Hence, approach around symbiosis is suggested as a disease therapy model as it is assumed that protozoa that inhabit naturally in human tissues can be genetically altered for the production and transfer of curative proteins. One of the examples for such kind of therapy model is *Leishmania* that has been genetically modified for conditional auxotrophy and indicated no disease-causing signs in non-human primate and mouse model-based clinical examinations. Numerous protozoan species have been transfected with a plethora of genes and have been observed producing active foreign proteins quite efficiently hence contributing their positive attribute on the medicinal front (Vaccaro, 2000).

#### 2.4.2 Protozoa in wastewater treatment

Wastewater treatment is considered a pivotal method in the context of the ever-increasing human population. Wastewater processing facilities are intended to manage higher activity and density measures of those microorganisms that actively participate in various purification methods. With context to this, protozoa are considered to be responsible for repairing the nature of the effluent as well as controlling the bacterial population by predation and hence are taken as one of the most pivotal parts in the man-made system environment and play a crucial role in wastewater purification. Investigations performed on ciliates considering acute toxicity of contaminants suggested protozoans as potential bio-indicators for measuring water toxicity contaminated by various metal doses (Madoni, 2011). One of the research investigations examined the bacterivorous behaviour of ciliates that were isolated from artificial wetlands using the root zone method of wastewater treatment. Examples of ciliates that were seen to graze on fluorescently labelled bacteria, most notably *Escherichia coli* include *Paramecium* spp. oxytrichids, *Halteria* and scuticociliates suggesting their positive role in wastewater treatment (Decamp and Warren, 1998). Additionally, it has been established that *Uronema marinum* is an indicator of eutrophication and situations that are both oxygen-poor and rich in organic matter, while algivorous ciliates like *Tintinnopsis baltica* and *Favella ehrenbergii* are indicators of low nutrient levels and bacterivorous/detritivorous environments (Ravindran et al., 2023).

## 3 Fungi

The realm of fungus is made up of a wide variety of fungal species that are highly diverse and distinct from one another. There are numerous dangerous fungal species throughout this enormous planet. The majority of them are infections that affect plants, but some of them can be fatal to humans, animals, and other living things. The fields of medical mycology and microbiology have integrated the study of these hyphal infections and are no longer considered to be distinct fields. Fungal diseases are extremely important for public well-being: More than 14 billion global cases are reported of which over 1.5 million annual deaths are related to fungal infections (Casadevall, 2007). A fungal infection primarily affects immunocompetent, healthy individuals. Complementarily, there has been a rise in the quantity of these patients experiencing catastrophic fungal infections.

### 3.1 Fungal infection dynamics

The body may be exposed to fungus through several methods such as inhaling them into the lung or taking them by eating or colonizing other surfaces. Such injuries of anatomic barriers allow for the colonization of certain susceptible persons with local diseases. For example, these main risk factors include HIV/AIDS, cancer, organ transplantation, inherited or acquired immune deficiencies and other immune-suppressive events. Invasive interventions, antibiotic usage, as well as pathogen sources, can give rise to nosocomial fungal infections that pose huge threats for hospitalized patients. Acute rapidly progressive fungal infection tends to be serious in terms of mortality, which is worse when compared with sub-acute and chronic infections. Therefore, the development of the disease is determined by the fungal virulence traits and the health of the host (Rokas, 2022).

### 3.2 Pathogenic mechanism in fungi by immune evasion mechanisms and other strategies

There are many ways by which the fungal pathogens have evolved strategies to dodge or manipulate the host immune system functioning. Mechanisms such as escaping into the inner spaces of certain cells like those in the immune system, silencing immune activation in addition to reprogramming of the host immune response (Maertens et al., 2002; Li et al., 2019).

#### 3.2.1 Adherence to the extracellular matrix components

One of the most important steps in the course of infection is thought to be binding on the host tissue, which appears to be facilitated by the identification of certain Extracellular Matrix components, such as basement membrane laminin, tenascin, types I and IV collagens, and fibrinogen. Disseminated infection most likely develops metastatic foci of infection throughout the body after attachment to the sub-endothelial extracellular matrix (ECM). In *Candida albicans* germinating cells, a variety of cell-surface receptors (68, 62, and 60 kDa) with various affinities for laminin, fibrinogen, and C3d were discovered. A 72-kDa cell-wall receptor and a 37-kDa laminin-binding receptor were identified in *Aspergillus fumigatus*. A glycoprotein of 120 kDa known as BAD1, is the most well-studied adhesin in *Blastomyces dermatitidis*. It can facilitate the adherence of yeast cells to host cells or extracellular matrix proteins. Thus, pathogenic fungal parasites can destabilize the molecules of their host to obtain entry that aids in their survival and development.

#### 3.2.2 Internalization into host

Entering eukaryotic cells allows harmful fungi to take advantage of the generally unsuitable environment as a place to proliferate or hide from the host immune system. *Candida albicans* can cause endothelial cells to undergo phagocytosis by polymerizing their microfilaments and microtubules. The actin cytoskeleton can undergo disorganization due to the direct influence of fungal products released by *Candida*, such as actin-rearranging Candida-secreted factor (ARCSF), acting on actin or associated proteins. This disorganization is subsequently accompanied by rearrangement of cellular actin, a decrease in membrane ruffling, and a decline in cell motility. Wasylnka and Moore conducted an *in vitro* study exploring the uptake of two distinct strains of *Aspergillus fumigatus* in A549 lung epithelial cells, human umbilical vein endothelial cells (HUVEC), and J774 murine macrophages. The cytoskeleton of the host cell had to be rearranged for A549 to internalize the conidia. These results show that non-professional phagocytes can internalize a significant number of *Aspergillus fumigatus* conidia *in vitro*, and these cells may serve as repositories for immune cell evasion and host-disseminated immunity.

#### 3.2.3 Regulation of pathogenicity via signaling mechanisms

The investigation of signal transduction pathways in pathogenic fungi holds particular significance due to their potential involvement in the control of pathogenicity. A wide range of cellular functions are regulated by cAMP signaling cascades in both pathogenic and non-pathogenic fungi. Adenylyl cyclase-dependent signaling pathways govern the development of certain phenotypes necessary for virulence in *Candida albicans* and *Candida fumigatus*. Strains of *Candida albicans* (CaCDC35) containing a mutation in the adenylyl cyclase gene are unable to go through the morphological transition from budding yeast to polarised form, which is required for the organism to reach its maximal virulence. By extending their membranes and eluding macrophage defenses, invasive *Candida albicans* yeasts form germ tubes inside phagolysosomes and swiftly invade macrophage late endosome and lysosome compartments. These pathways may aid in the pathogen’s survival and pathogenicity. It appears that the uptake of *Candida* yeasts depends on protein kinase C (PKC) activity, needing intact actin filaments, and hence exhibiting phagocytosis-like features (Mendes-Giannini et al., 2005).

### 3.3 Fungal infection in humans

#### 3.3.1 Aspergillosis

*Aspergillus*, a prevalent mold (a specific kind of fungus) that can flourish both indoors and outdoors is the major underlying cause of the disease aspergillosis. *Aspergillus*-related health issues are more likely to develop among individuals with compromised immune systems or respiratory disorders. Aspergillosis occurs in various forms, while some varieties are minor, others are extremely infectious (Barnes and Marr, 2006). About 8 different types of aspergillosis are described hereunder including:

- Chronic pulmonary aspergillosis: Occurs when lung cavities brought on by *Aspergillus* infections persist for 3 months or longer. There may also be the presence of aspergillomas (fungal balls) in the lungs (Denning et al., 2003; Graham and Nasir, 2019).
- Aspergilloma (Fungus Ball): It is caused by an *Aspergillus* ball that grows in the sinuses or lungs and often does not spread to the other body regions (Latgé, 1999).
- Allergic *Aspergillus* sinusitis: Occurs when *Aspergillus* stimulates the sinuses and produces signs of infection including headache, stuffiness and drainage without actually causing the infection (Panjabi and Shah, 2011).
- Allergic bronchopulmonary aspergillosis (ABPA): In this case, *Aspergillus* irritates the lungs and generates allergic symptoms like coughing and wheezing (Rajpopat et al., 2022).
- Invasive aspergillosis: It typically affects individuals with compromised immune systems or those individuals who have undergone organ or stem cell transplantation. This type of aspergillosis generally affects the lungs however, it can also spread to other body regions as well (Dagenais and Keller, 2009).
- Azole-Resistant *Aspergillus fumigatus*: Occurs when *A. fumigatus*, a type of *Aspergillus*, develops resistance to a number of medications used to treat it (Burks et al., 2021).
- Cutaneous (skin) aspergillosis: In this condition, *Aspergillus* penetrates the body through an opening in the skin and produces infection. If invasive aspergillosis originates from any other part of the body, like the lungs and spreads to the skin then it also results in cutaneous aspergillosis (van Burik et al., 1998).

##### 3.3.1.1 Clinical features

Symptoms associated with various types of aspergillosis include cough, shortness of breath and wheezing (allergic bronchopulmonary aspergillosis), decreased smelling ability, stuffiness and headache (allergic *Aspergillus* sinusitis) (Roth and Schatz, 2013). Signs associated with aspergilloma involve coughing up blood and shortness of breath (Lee et al., 2004), whereas patients infected with chronic pulmonary aspergillosis display indications such as weight loss, shortness of breath, fatigue as well as coughing of blood in response to the infection (Denning et al., 2003; Schweer et al., 2014). Moreover, hemoptysis, dyspnea, persistent productive cough, and chest pain are also observed as common symptoms in chronic pulmonary aspergillosis patients. All forms of chronic pulmonary aspergillosis cause hemoptysis, which occasionally progresses to potentially fatal large hemoptysis (Hou et al., 2017). Apart from this, invasive aspergillosis can cause cavitation, nodules, gradual consolidation, and the formation of an abscess. This condition is typically seen in patients who have become only slightly immunocompromised (Raveendran and Lu, 2018).

##### 3.3.1.2 Pathogenesis

About 90% of cases of invasive Aspergillosis are caused by the opportunistic pathogen *Aspergillus fumigatus*, which carries a notably high fatality rate. The primary mechanisms by which *A. fumigatus* becomes pathogenic to the host encompass direct infection with virulent factors of the pathogen, individual hypersensitivity reactions, or activation of the host’s innate and adaptive immunity due to virulent factors when the pathogen colonizes the host. Over the past few decades, numerous factors have been associated with *A. fumigatus* virulence, including thermotolerance, cell wall composition and integrity, resistance to immune responses, production of toxins, nutrient acquisition during invasive growth, regulation of signaling pathways, and allergenic properties. This understanding notably advanced following the sequencing of the *A. fumigatus* AF293 strain’s genome in 2005. Given its thermophilic nature, *A. fumigatus* can thrive at temperatures up to 75°C and proliferate particularly well at 55°C. This characteristic enables it to colonize and grow in decomposing or decaying organic matter and to infect mammalian host cells more effectively. Consequently, *A. fumigatus* pathogenicity is partially attributed to genes associated with thermotolerance, including thtA, cgrA, afpmt1, kre2/afmnt1, and hsp1/aspf12. Additionally, toxins produced by *A. fumigatus* can directly harm the host and contribute to the fungus’s pathogenesis, providing defense against predators. Many of these toxins are fungal secondary metabolites that can alter cell membranes, hinder cellular functions, or affect the synthesis of proteins, RNA, and DNA. Numerous toxins and associated genes of *A. fumigatus* have been studied, including the transcription factor laeA, diffusible toxic compounds from conidia, gliotoxin (gliP and gliZ), mitogillin (res/mitF/aspf1), hemolysin (aspHS), fumagillin, and verruculogen. Among these, gliotoxin stands out as the most potent toxin produced by *A. fumigatus*, capable of inhibiting monocyte apoptosis, T cell proliferation, cytotoxic T cell response, and macrophage phagocytosis (Gu et al., 2021).

##### 3.3.1.3 Diagnosis and control

Histopathology, culture and direct microscopy ideally with optical brighteners are strongly suggested for diagnosis. Usage of serum and bronchoalveolar lavage galactomannan measurements is strongly advised as prominent diagnostic indicators for pulmonary invasive aspergillosis. Apart from this, it is highly advised that all clinically relevant *Aspergillus* isolates have their infection identified down to the species complex level. For the first line therapy of Pulmonary invasive aspergillosis, voriconazole and isavuconazole are recommended (Ullmann et al., 2018). Some of the control measures suggested for the prevention of aspergillosis include-.

- Protection from the surroundings by avoidance of tasks like gardening or yard work that require close contact with dirt or dust, usage of gloves when working with things like dung, moss or soil and cleaning skin injuries well with soap and water to lessen the possibility of developing a skin infection (Ullmann et al., 2018; Avery et al., 2019).
- Providing antifungal medication to individuals who have undergone stem cell or organ transplantation as they develop elevated risk towards invasive aspergillosis (Panackal et al., 2014).
- Blood testing may also prove to be useful for certain high-risk patients to identify invasive aspergillosis (Barton, 2013).

### 3.4 Beneficial role of fungi

#### 3.4.1 Role of beneficial fungus in sustainable farming

Fungi is one of the most essential classes of microorganisms that could potentially be used in a variety of industries, including agriculture. Valuable fungi play a significant part in the long-term viability of agriculture (Watts et al., 2023). These fungi come in a variety of forms, including symbiotic and endophytic varieties. The potential of beneficial fungi could be used as an alternative to chemical pesticides for the management of plant pathogens, pests and weeds. Endophytic fungi, mushrooms, entomopathogenic fungi and dark septate fungi fall among the well-known groups of beneficial fungi. Certain beneficial fungi are also observed to be associated with the enhancement of plant growth. The beneficial effects of using fungi in agriculture encouraged the industries to use them to create biopesticides and biofertilizers which can further be used in place of synthetic chemicals and reduce the amount of chemical waste in the environment. Hence, fungi play a significant role in sustainable farming by gaining incorporation into integrated pest and disease management strategies (Lavado and Chiocchio, 2023).

#### 3.4.2 Diagnostic approaches for fungal infections

There are various methods by which diagnosis of fungal pathogens can be established (Fang et al., 2023). Table 2 represents various strategies involved in the detection of infections caused by disease-causing fungi.

**Table 2:** Representing various advantages and disadvantages associated with diagnostic approaches used for detection of fungal infections.

Diagnostic approach	Advantages	Disadvantages
Clinical evaluation	Rapid and non-invasive	Subjective and may not be specific for fungal infection
Direct microscopy	Rapid and inexpensive	May not be sensitive enough to detect all fungal infections
Fungal culture	Gold standard for species identification and antifungal susceptibility testing	Time-consuming (may take several days to weeks)
Molecular diagnostics	Highly sensitive and specific	Expensive and requires specialized equipment
Serological tests	Non-invasive and can be used to diagnose systemic fungal infections	May not be sensitive enough to detect early infections or in immunocompromised patients
Radiological imaging	Can be used to diagnose invasive fungal infections affecting internal organs	May not be specific for fungal infection
Skin tests	Can be used to diagnose specific fungal infections and determine exposure	Not sensitive enough to diagnose active infection and may give false-positive results in patients with prior exposure
Endoscopy and biopsy	Definitive diagnosis of invasive fungal infection	Invasive and may carry risks
MALDI-TOF mass spectrometry	Rapid and accurate identification of fungal isolates	Requires specialized equipment and not widely available
Next-generation sequencing	Detailed genomic information about fungal pathogens, allowing for precise species identification and detection of antifungal resistance markers	Expensive and requires specialized equipment

#### 3.4.3 Antifungal therapies

**Figure 3:**
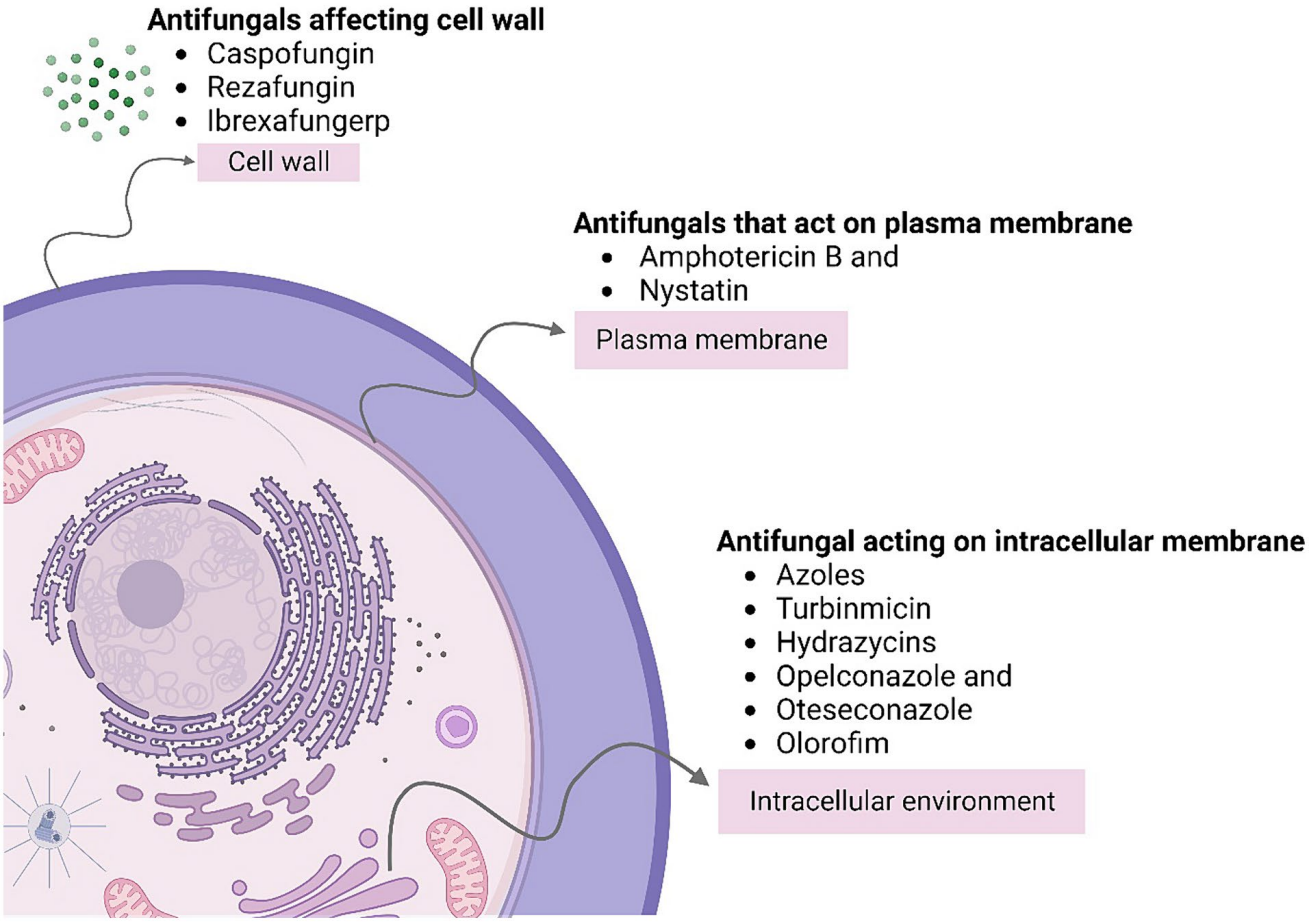
Describes the types of antifungal agents acting on different sections of the cell causing prevention of fungal infection.

Antifungal drugs interfere with various aspects of fungal cell biological function, thus preventing further growth of the pathogen (Sanguinetti et al., 2015). Figure 3 describe the types of Antifungal Agents acting on different sections of the cell causing prevention of fungal infection these drugs can be classified into several categories described as:

- Azoles: Azoles like fluconazole and itraconazole inhibit the production of ergosterol, which is an important product for the cellular barrier in fungi. Primarily, these agents work well for superficial or systemic fungus infections.
- Echinocandins: Echinocandins include drugs like caspofungin and micafungin which hinder the creation of beta-1,3-D-Glucan, a component of the cell wall found in fungi. It has been proved that these types of antibiotics help in the treatment of almost all *candidal* and *aspergillus* species infections.
- Polyenes: The binding of the polyene antifungal agent, such as amphotericin to the ergosterol in the fungal cell wall leads to permeability disruption of the latter. Hence, preventing the cycle of the infection (Sanguinetti et al., 2015).

## 4 Worms

Worms are a diverse group of creatures that include helminths and nematodes, which can parasitize humans, animals, and plants. Over a billion people worldwide suffer from serious parasitic ailments, which have a devastating impact on morbidity and mortality. Worms have also proven to be useful as pivotal model structures for investigating host–parasite interactions, and targeted treatments in addition to the mechanisms associated with the disease. This section demonstrates the depth of understanding offered by the worm models such as *Schistosoma mansoni* and *Caenorhabditis elegans*, that provide insights into virulence factors, host immune responses, and pathways for intervention against both microbial pathogens and parasitic worms (Derakhshani et al., 2022).

### 4.1 Worms as esteemed model organisms

**Figure 4:**
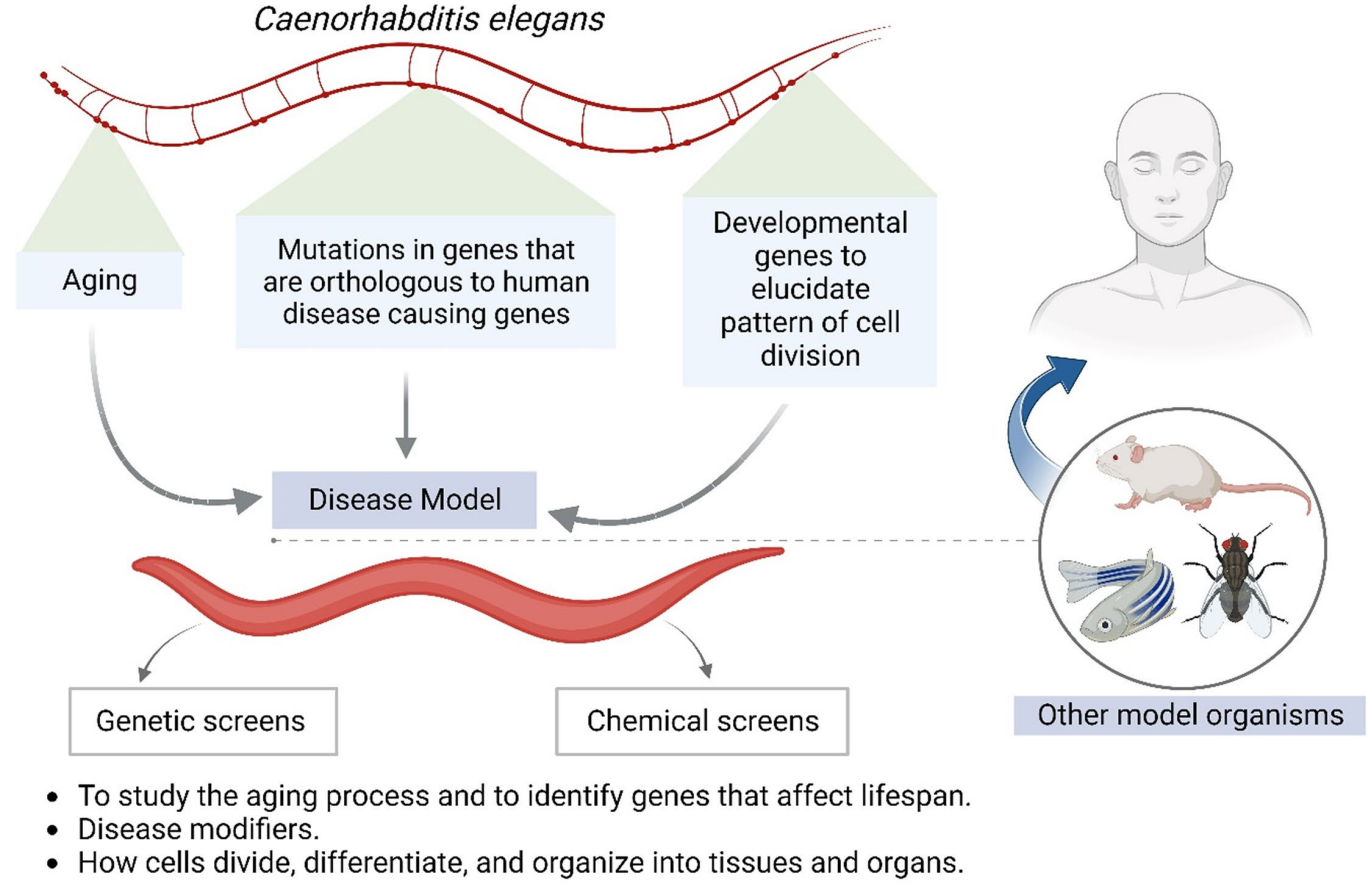
Shows *Caenorhabditis elegans* as a model system for understanding human associated maladies.

*C. elegans* has emerged as one of the most powerful model organisms in biology due to its rapid life cycle, ease of laboratory manipulation, simple anatomy, and wealth of genetic tools. A large number of studies have utilized *C. elegans* for pivotal discoveries related to developmental biology, neuroscience, ageing, and host-pathogen interactions. Figure 4 shows *Caenorhabditis elegans* as a model system for understanding human-associated maladies. Its transparent body enables unprecedented visual dissection of infection processes. Researchers have even crafted mini-biosuits to explore worm behavioural responses to pathogens. Among helminths, *S. mansoni* stands out given its vast clinical impact and toolbox of research resources. Chronic schistosomiasis afflicts over 200 million people, underscoring the need for better therapies. *S. mansoni* can readily infect mice, facilitating detailed molecular investigations. Importantly, its intricate multi-cellular structure provides closer similarity to human parasites than single-celled models. Cross-species omics studies have revealed deep conservation of pathways regulating worm development, making *S. mansoni* invaluable for the identification of new intervention targets.

### 4.2 Diverse immune strategies against worms

Worm pathogens interface in detail with host immunity in a multifaceted manner. Infection elicits inflammation mediated by cytokines like IL-5, eosinophilia, mucus manufacturing, and IgE. CD4 T cells orchestrate immune mobilization and granuloma formation to isolate worms. However, hosts face challenges in removing worms absolutely because of their elusive nature and immunomodulatory secreted products. For example, filarial nematodes secrete phosphorylcholine-containing glycoproteins that result in IL-10 and modify dendritic cells to facilitate patience. Tropical worms express novel chemokine binding proteins that selectively goal key chemokines to undermine the recruitment of herbal killer cells and T cells. The excretory-secretory antigen Omega-1 from *S. mansoni* eggs controls IL2 production by way of CD4 T cells to selectively hose down Th2 responses. These cases illustrate sophisticated co-evolutionary adaptations of worms to guide host immunity (Mouser et al., 2019).

### 4.3 Pathogenic mechanism in worms

#### 4.3.1 Penetration in the host organism through parasitic secretions

The term “elastases” refers to the soluble serine proteases of *Schistosoma mansoni* cercariae. The transcripts of at least three of these serine proteases have been found in the precursor germ balls of the daughter sporocyst stage in the snail host, and they have been biochemically characterized from the secretions of the acetabular gland. The proteins of the stratum corneum and the epidermis, where the entering cercarial body forms a penetration tunnel, are most likely the substrate of the characterized serine proteases, and as the parasite penetrates the epidermis, its thick protective glycocalyx and cercarial tail are shed. Glycans are another component of cercarial secretions that is worthy of consideration as a virulence factor. The proteins found in soluble cercarial secretions have high levels of glycosylation. Mass spectrometry has discovered a large number of both N-and O-linked glycan structures, many of which are shared between the two stages of transmission, indicating a shared function by the stages entering and exiting the host. Therefore, as a component of the immune evasion system, glycans act as a covert barrier to divert antibodies from functional peptide epitopes that are susceptible to attack, drawing leucocytes away from the approaching larva to let them escape from the protective host machinery.

#### 4.3.2 Parasite’s tegument and immune evasion

A syncytial layer of cytoplasm connects the schistosome interface with the circulation, often known as the tegument, to cell bodies located beneath the muscle. Evasion of the host immune response primarily takes place on the surface of the tegument. The ability of a few exposed enzymes on the outer leaflet of the tegument plasma membrane to alter the parasite’s immediate environment within the blood vessel has led to their being considered as possible virulence factors. The discovery that ATP-diphosphohydrolase functioned externally on the tegument raised the possibility that the enzyme may control the number of purine nucleotides surrounding the parasites, allowing them to evade the host’s hemostasis by blocking ADP-induced platelet activation. It has also been observed that the external surface GPI-anchored ADP-ribosyl cyclase enzyme may catabolize extracellular NAD+ to stop host enzymes from using it to promote immunological responses. Additionally, tegumental alkaline phosphatase’s production of adenosine from adenosine monophosphate has been proposed to have a localized immunosuppressive effect (Wilson, 2012).

### 4.4 Diseases in humans

#### 4.4.1 Fasciolosis (liver fluke)

Fasciolosis is an extremely infectious parasitic disease that infects humans as well as livestock and is caused by flat worms that belong to the genus *Fasciola. Fasciola hepatica* belongs to class trematoda and the infection caused by it is responsible for causing serious mortality and morbidity and is associated to elevated sensitivity to co-infections and decreased yield and potency. Consuming infected plants and vegetables is the primary way that humans acquire liver fluke infection. *F. hepatica* uses snails as intermediary hosts in its life cycle. When the pathogen finds appropriate environmental conditions, the meracid undergoes the asexual phase of its life cycle after infecting the snail host. Eventually, it transforms into cercariae (Lalor et al., 2021).

##### 4.4.1.1 Clinical features

There are two clinical phases of fascioliasis: acute and chronic. Signs and symptoms are contingent upon the phase, duration, and burden of worm infection. It is possible to detect eosinophil absence in the very early stages of acute illness. Few days later, an abrupt increase in the eosinophil count can be observed. When fascioliasis is highly suspected, it may be appropriate to repeat a blood test for eosinophil count 3 to 5 days later. Additional symptoms include lymphadenopathies, arthralgias, anorexia, weight loss, nausea, vomiting, coughing, diarrhea, and urticaria. When the parasite enters the bile ducts, the chronic phase starts. The majority of patients do not display any symptoms. When symptoms appear, they include urticaria, jaundice, nausea, vomiting, and recurring stomach discomfort in the right upper quadrant. All of these indicate biliary obstruction. At this stage, the liver has big, calcareous, dilated bile ducts that contain bile that is yellowish-brown in colour (Caravedo and Cabada, 2020).

##### 4.4.1.2 Pathogenesis and host defense

Numerous factors influence the infectivity of metacercariae, including the ultimate host, parasite strain, climate, seasonal variations, snail species acting as hosts, and the developmental stage of snail larvae. As metacercariae represent the infective stage of *Fasciola* spp., the quantity of metacercariae ingested, the strain’s isolation, and the type of host all play roles in the disease’s progression. Shortly after ingestion by the mammalian host, metacercariae undergo excystment, and newly excysted juveniles (NEJs) promptly starts penetrating the host intestinal wall. In the stomach, elevated CO2 levels and a temperature of approximately 39°C trigger larval activation within the inner cyst. The upregulation of genes associated with cytoskeletal proteins such as talins and cell adhesion molecules like integrins and cadherins in metacercariae likely occurs due to their ability to sense environmental cues necessary for initiating the excystment process. NEJs release a plethora of virulence-associated compounds, including proteolytic enzymes and stage-specific peptidases, which degrade extracellular matrix (ECM) components and maintain tissue integrity. Five cathepsin cysteine peptidases, specifically cathepsin L3 (FhCL3) and cathepsin B peptidases (FhCB1, FhCB2, FhCB3, and FhCB9), exhibit high expression and subsequent excretion-secretion, facilitating rapid excystment of metacercariae and eventual invasion by NEJs into the host. Research indicates that these peptidases are essential for the parasite’s pathogenicity (Lalor et al., 2021).

When comparing the pathogenesis to the infection level, the trauma-like stage produced by the juvenile flukes sifting through the liver parenchyma and intestines is largely associated. The symptoms of the acute stage may be mild or completely absent, but later on, they may develop into a complex chronic inflammatory condition. However, acute-stage infections are represented by active immune response towards the pathogenic antigens and are observed in the form of anaemia, fever, abdominal pain, hepatomegaly, transitional eosinophilia and increased levels of liver-associated enzymes With context to *F. hepatica* infection, Th2 response is observed majorly with short span of early Th1 response that is suggested to be functioning against the infection caused by the fluke. In case of animals having susceptibility to the parasite (for example sheep), a mixed reaction is observed considering Th1 and Th2 with IFNγ and IL-10 production.

##### 4.4.1.3 Diagnosis and control

Human fasciolosis is relatively easy to diagnose in endemic places because the disease is sporadic there, but it might be difficult to diagnose in areas where the infection is rare. Diagnostic hallmarks observed in the patients include headache, fatigue, chills, sweats, abdominal pain as well as rashes. Apart from this, in order to detect hypodense liver nodules and lesion computerized topography (CT) is performed. In major duodenal papilla by utilizing endoscopic retrograde cholangiopancreatography live worms are visualized. Since no vaccine is available to provide protection from the infection, control on the individual level is suggested. Avoiding eating water plants and practicing good hygiene measures could be adopted to protect oneself from the infection (Morales et al., 2021).

### 4.5 Beneficial role of worms

#### 4.5.1 Maintenance of soil fertility

All ecosystems benefit from the contribution of the soil biota to soil production and long-term viability. Earthworms (EWs) make up a significant amount of the biomass of macrofauna and are a major component of soil fauna communities in most habitats. Their activity is advantageous because it can improve the nutrient cycle of soil by quickly incorporating detritus into mineral soils. Apart from the effect of mixing, the creation of mucus resulting from water excretion in the stomachs of earthworms also amplifies the activity of other advantageous soil microbes. Furthermore, earthworms appear to quicken the process of soil organic matter turnover and mineralization. Through their direct and indirect effects on the microbial population, earthworms are also known to boost nitrogen mineralization. The potential for earthworms to aid in the stabilization and accumulation of soil organic matter in agricultural systems is shown by the increased transfer of organic carbon and nitrogen into soil aggregates (Bhadauria and Saxena, 2010). Some of the examples of earthworms that aid in the improvement and maintenance of soil fertility include *Hyperiodrilus africanus* and *Eudrilus eugeniae* (Tian and Badejo, 2001).

#### 4.5.2 Vermicomposting

Earthworms and other microorganisms break down and stabilize organic waste through a process called vermicomposting. The organic waste substrates are broken up by the earthworms, which also significantly boosts microbial activity and accelerates mineralization rates. Since vermicompost is a stable, fine-granular organic matter, adding it to clay soil enhances airflow and loosens the soil. The mucus connected to the hydrophobic cast enhances water-holding capacity by absorbing water and preventing water logging. The plant can absorb the nutrients because of the organic carbon in vermicompost, which distributes the nutrients into the system gradually. Additional nutrients that are absent from artificial fertilizers are added to the soil when it is treated with vermicompost. Vermicomposting provides a way to recycle and utilize the tonnes of organic agricultural waste that farmers are burning to support our agricultural development in a more economical, ecologically friendly, and efficient way. Earthworms play a well-established function in the management of organic solid waste, and waste products can be processed using adapted technology to create vermicompost, an effective bioproduct. Vermicomposting is done with epigeic earthworms such as *Perionyx excavatus*, *Eisenia fetida*, *Lumbricus rubellus*, and *Eudrilus eugeniae*; however, native species, such as *Perionyx excavatus*, have shown to be effective composting earthworms in tropical or sub-tropical environments (Garg et al., 2006; Ali et al., 2015).

## 5 Conclusion and future prospects

Our knowledge of the intricate relationships that exist between hosts and pathogens has been expanded as a result of host-pathogen investigations. The complex relationship between the immune system and invasive microbes has been a focus, offering insights into immune responses, infection processes, and pathogen evasion tactics. Hence, the information provided in this review can further help in providing ideas towards better exploration of this domain in a more targeted way. In summary, there are a number of promising avenues for future research in host-pathogen investigations, including personalized medicine, vaccine development, microbiome treatments, systems biology techniques, and combating antimicrobial resistance which in turn will help in enhancing global awareness for infectious threats.

## Author contributions

RS: Visualization, Writing – original draft. JS: Visualization, Writing – review & editing. AK: Investigation, Writing – original draft. RP: Conceptualization, Funding acquisition, Project administration, Supervision, Visualization, Writing – review & editing.
